# The comparative effectiveness of metformin and risperidone in a rat model of valproic acid-induced autism, Potential role for enhanced autophagy

**DOI:** 10.1007/s00213-023-06371-1

**Published:** 2023-05-03

**Authors:** Amany AA Atia, Rehab H Ashour, Marwa MAF Zaki, Karawan MA Rahman, Nehal M Ramadan

**Affiliations:** 1 Department of Clinical Pharmacology, Faculty of Medicine, Mansoura University, 60 El-Gomhoria Street, Mansoura, Al-Dakahlia 35516 Egypt; 2 Department of Pathology, Faculty of Medicine, Mansoura University, 60 El-Gomhoria Street, Mansoura, Al-Dakahlia 35516 Egypt

**Keywords:** ASD, Metformin, Risperidone, autophagy, LC3B, P62

## Abstract

**Rationale:**

Risperidone is the first antipsychotic to be approved by Food and Drug Administration (FDA) for treating autism spectrum disorder (ASD). The potential efficacy of metformin in preventing and/or controlling ASD behavioral deficits was also recently reported. Suppression of hippocampus autophagy was suggested as a potential pathologic mechanism in ASD.

**Objectives:**

Is metformin’s ability to improve ASD clinical phenotype driven by its autophagy-enhancing properties? And does hippocampus autophagy enhancement underlie risperidone’s efficacy as well? Both questions are yet to be answered.

**Methods:**

The effectiveness of metformin on alleviation of ASD-like behavioral deficits in adolescent rats exposed prenatally to valproic acid (VPA) was compared to that of risperidone. The potential modulatory effects of risperidone on hippocampal autophagic activity were also assessed and compared to those of metformin.

**Results:**

Male offspring exposed to VPA during gestation exhibited marked anxiety, social impairment and aggravation of stereotyped grooming; such deficits were efficiently rescued by postnatal risperidone or metformin therapy. This autistic phenotype was associated with suppressed hippocampal autophagy; as evidenced by reduced gene/dendritic protein expression of LC3B (microtubule-associated proteins 1 light chain 3B) and increased somatic P62 (Sequestosome 1) protein aggregates. Interestingly, compared to risperidone, the effectiveness of metformin in controlling ASD symptoms and improving hippocampal neuronal survival was well correlated to its ability to markedly induce pyramidal neuronal LC3B expression while lowering P62 accumulation.

**Conclusions:**

Our work highlights, for the first time, positive modulation of hippocampus autophagy as potential mechanism underlying improvements in autistic behaviors, observed with metformin, as well as risperidone, therapy.

## Introduction

Autism Spectrum Disorder (ASD) is a lifelong Neuro-developmental disability manifesting in two key behavioral symptoms: persistent impairment of social reciprocal interaction/communication, and restricted, repetitive/stereotyped patterns of behavior or interests (American Psychiatric Association 2022). With a male to female ratio of 4:1 (Dana et al. 2020), ASD symptoms begin to show up in the first year of life and can be clinically detected between 6 and 18 months of age (Tanner and Dounavi 2021). Diagnosis of ASD remains extremely challenging owing to the wide diversity of its clinical phenotypes, co morbidities and numerous levels of severity (Bougeard et al. 2021)**.** That is possibly the reason why high variability in ASD prevalence was reported across different regions; 0.42-3.13% in Europe, 0.11-1.53% in Middle-East, 0.08-9.3% in Asia, and 0.87-1.85% in North America (Chiarotti and Venerosi 2020).

Effective control of the core behavioral abnormalities of ASD is yet waiting for therapy. The two antipsychotic medications currently approved by Food and Drug Administration (FDA) to treat ASD, aripiprazole and risperidone, possess moderate efficacy, while principally targeting the accessory anxiety and aggressiveness (Fieiras et al. 2022). The ability of risperidone to improve ASD-like social deficits was not consistent across the wide panel of preclinical models of ASD. While it was effective in ASD models, as Cntnap2 knockout mice (Peñagarikano et al. 2011) and BTBR mice (Amodeo et al. 2014), it lacked efficacy in NMDA receptor NR1 subunit knockdown mice (Teng et al. 2016). However, Via largely unclear mechanisms, evidence stemming from valproic acid (VPA)-induced ASD rat model has repeatedly supported effectiveness of risperidone therapy (Hara et al. 2017; Román et al. 2021; Elnahas et al. 2022).

Developing/repurposing pharmacological treatments, to target early developmental abnormalities related to ASD, represents an effective approach towards control of ASD core symptoms. Metformin, the dimethylbiguanide that was isolated in the twenties of the last century, from the extract of French lilac flowers, is now the first-line anti-diabetic drug worldwide (Bailey 2017). Deeper understanding of metformin’s mechanism of action, along with its ability to cross the blood brain barrier has fueled research of repurposing this considerably safe drug for treatment of many neuro-psychiatric disorders. Not only showed efficacy against diabetes-related memory (Mousavi et al. 2015) and mood deficits (Hsu et al. 2011), metformin was also reported to confer neuroprotection against apoptotic cell death in primary cortical neurons (Ullah et al. 2012); rescue spatial memory impairment and promote hippocampal neurogenesis in Alzheimer’s disease (Liao et al. 2022); rebalance neuronal aberrations in a model of Huntington’s disease (Arnoux et al. 2018); attenuate neuronal loss and improve locomotor and muscular activity in models of Parkinson's disease (Mor et al. 2020). In the context of ASD, it was shown to improve the core cognitive and behavioral phenotypes in patients with fragile X syndrome (FXS) (Dy et al. 2018), along with mediating metabolic improvement and control of antipsychotic-induced weight gain in adolescents with ASD (Anagnostou et al. 2016). Evidence suggests that neural AMP-activated protein kinase (AMPK) activation can readily explain metformin’s neuroprotective effects, such as preserving cellular energy homeostasis; promoting mitochondrial biogenesis; and enhancing brain-derived neurotrophic factor expression (Zhu et al. 2018). Additionally, autophagy induction, downstream of AMPK activation and/or mammalian target of rapamycin (mTOR) inhibition, was implicated in the metformin neuroprotective properties (Demaré et al. 2020).

Autophagy is the cellular catabolic process dedicated for degradation and recycling of aberrant organelles and damaged proteins (Mizushima et al. 2008). Relevant literature has proposed a general causal link between neuronal autophagic dysregulation and pathogenesis of ASDs (Lee et al. 2013). Of note is the previously suggested role of reduced brain autophagy in the development of ASD-related social impairments characteristic of prenatal exposure to VPA (Zhang et al. 2016). These results have prompted us to hypothesize that metformin and, possibly, risperidone may confer alleviation of the social and repetitive behavioral deficits of ASD through their autophagy enhancing properties.

Using behavioral, biochemical and immunohistochemical approaches, this study highlights for the first time the enhanced hippocampal autophagic activity as a principal mechanism of improvement of autistic rats on either risperidone or metformin therapy.

## Methods

### Chemicals and antibodies

The following drugs were purchased in the form of powder from Sigma-Aldrich Co. (St. Louis, MO, USA): Sodium Valproate (VPA, 2-propylpentanoic acid, sodium salt, CAS Number 1069-66-5); Metformin Hydrochloride (CAS Number 1115-70-4); Risperidone (CAS Number 106266-06-2). The following reagents were purchased: rabbit polyclonal anti-P62 antibody (AB clonal, USA); rabbit polyclonal anti-LC3B (Biospes, China); Horseradish peroxidase (HRP)-conjugated anti-rabbit IgG (Santa Cruz, USA); GeneJET RNA Purification Kit (Thermo Scientific, IL, USA); Super Script III First Strand cDNA Synthesis Kit (Thermo Scientific, IL, USA); SensiFAST™ SYBR® No-ROX Kit (Bioline USA Inc. USA); LC3B and beta-actin forward and reverse primers (Biosearch technologies, CA, USA). All other chemicals used were of high analytical grade.

### Animals

Thirteen adult breeding Sprague-Dawley pairs (200-250g) were purchased from the animal facility of Medical Experimental Research Center, MERC (Mansoura, Egypt). Rats were housed in standard cages under controlled temperature (22 ± 2°C), humidity (50 ± 5%) and 12:12 h light: dark cycle, with free access to water and food. Animals were handled according to “Guide for the Care and Use of Laboratory Animals” formulated by the institute of Laboratory Animal Research and published by the National Research Council, USA, 2011 (“Guid. Care Use Lab. Anim.,” 2011).

The study protocol was approved by Mansoura Faculty of Medicine-Institutional Review Board (MFM-IRB) under the code of MS.19.02.500 and all efforts were made to minimize animal numbers and suffering.

### Experimental design

According to the method of Schiavi et al., (2019), adult female rats were mated overnight, followed on the next morning by examination of their vaginal smear under light microscope. Females with vaginal plugs or sperms in their vaginal swap were affirmed as pregnant in the 1^st^ gestational day, E1. On E12.5, pregnant females received a single IP injection of either VPA (500 mg/kg, dissolved in 2ml isotonic saline) or saline. Litters born up to the 17:00 h in any given day were considered in their zero postnatal day, P0. All pups were allowed to grow till weaning, P21, when male offspring were separated and grouped as follows: control group (n=6) born to mothers injected with saline and treated with vehicle (0.5% v/v methylcellulose); VPA-untreated group (n=8) born to mothers injected with VPA and treated with vehicle (0.5% v/v methylcellulose); VPA-risperidone group (n=8), born to mothers injected with VPA and treated with oral risperidone (0.5 mg/kg/day suspended in 0.5% v/v methylcellulose) (Zhu et al., 2014); VPA-metformin group (n=8) born to mothers injected with VPA and treated with oral metformin (200 mg/kg/day suspended in 0.5% v/v methylcellulose) (Ishola et al. 2020). Vehicle and drug suspensions were administered by gavage once a day using a volume of 5 ml/kg. Two male pups per litter were randomly assigned to each experimental group to avoid the litter effects. The main reason for preferentially using male offspring is to eliminate the influence of sex differences on the results. That was based on the notion raised in our preliminary work (as well as previous similar studies (Kim et al. 2013; Melancia et al. 2018; Scheggi et al. 2020) that VPA-exposed male offspring consistently show significant impairment in social interaction indices compared to the female offspring; which show only marginal deficits in social interaction.

All rats survived the experimental period from P21 to P51 and no mortality was observed. It is agreed that early interventions targeting ASD core social impairments are crucial to affect the long-term outcome of the disorder (Burnside et al. 2017). We, and others, aimed to target a critical period of neurodevelopment (from P21 to P5) when regulating aberrant autophagic activity might rectify neurodevelopmental plasticity to rescue autism-like behaviors.

Six to eight rats per group underwent behavioral testing after completion of drug therapy, P52-P58; the three-chamber social approach test (P52-53), the spontaneous grooming test (P55-56) and the light/dark test (P58) with a one-day interval between each test.

### Behavioral Tests

Animals were handled throughout the whole study by the same researcher who performed all behavioral tests during the light phase (between 10:00 and 17:00 h). Different arenas were cleaned with 10% ethanol and allowed to dry between each two different animals. Whenever applicable, experiments were video-recorded by a high-resolution camera, and videos were analyzed by a researcher, who was blinded to the groups and treatments during analysis.

#### Three-chamber social approach test

Rodent sociability was assessed as previously described (Bambini-Junior et al. 2014; Wang et al. 2018), using the three-chamber apparatus (a clear glass rectangular apparatus (60 × 40 x 22 cm) with three equal chambers (20 × 40 × 22 cm). For habituation phase, the test rat was placed in the central chamber and allowed to freely explore the whole arena for 10 min. After habituation, a novel object (an empty mesh-wire container of suitable size) was placed in one of the lateral chambers, and a set of novel animal + mesh-wire container was placed in the opposite lateral chamber. The novel animal was an experimentally naive male rat of the same age, sex and strain as the test rat. During testing phase, the test rat was allowed another 10 min to interact with either the novel caged animal on one side, or stay on the other side with the novel object (empty container). Time spent in each chamber sniffing, and the number of entries into each chamber were analyzed. Preference index was calculated as follows:

$$\frac{Sniffing\ time\ in\ novel\ rat\ chamber- sniffing\ time\ in\ novel\ object\ chamber}{total\ sniffing\ time\ in\ both\ chamber s}$$

#### Spontaneous grooming test

To assess existence and severity of abnormal repetitive/stereotyped behaviors, rat self-directed grooming was analyzed, as described by Wang et al., (2018). Rats were individually habituated to a clean clear suitable Plexiglas chamber for 10 min, then video-taped for another 10 min to analyze their grooming activity. The following variables were calculated; duration of all self-grooming activities, number of complete grooming bouts and percentage of incorrect transitions (Table 1).

$$\%\ of\ incorrect\ transitions=\left(\frac{no.\ of\ incorrect\ ransitions}{Total\ no.\ of\ transitions}\right)\ast 100$$

**Table 1:** Calculated grooming variables

Complete grooming bout	An in-order grooming from 0-5 (0, no movement; 1, paw licking; 2, head wash; 3, body groom; 4, leg licking; 5, tail/genital licking), divided by at least 6 sec of inactivity or any other activity
Transitions	Total number of transfers between grooming movements
Incorrect transitions	Transfers between grooming movements which do not follow cephalo-caudal direction, ex. 1-3, 4-1

#### Light/dark (LD) transition test

Following the method previously described by Castelhano-Carlos et al., (2014), the anxiolytic effects of metformin and risperidone were compared using LD test. This test makes use of the conflict between innate rodent drive to explore novel areas and their aversion of brightly lit, open spaces. Briefly, the test box (51 length x 51 width x 40 cm height) consisted of one larger lit compartment (51 × 34.5 × 40 cm) illuminated by a white fluorescent lamp (400 lux at the box floor), and a smaller dark compartment (51 × 16.5 × 40 cm). The two compartments were connected via a small opening (7.5 cm × 8.5 cm). Rats were placed in center of the light compartment, facing the opening to dark, and were allowed to freely explore the arena for 5 min. For characterizing anxiety level of animals in each group, the time spent in light compartment, as well as, the number of transitions between the two compartments were recorded.

### Brain tissue sampling protocol

On P59, animals were euthanized with an overdose of sodium pentobarbital (200 mg/kg IP) (Zatroch et al. 2017), followed by transcardiac perfusion with 40 ml phosphate buffered saline (PBS, pH 7.4). Brains were rapidly excised, rinsed in ice-cold PBS and divided mid-sagittally into two hemispheres. The hippocampi were dissected from the ventral cortex where; the right-sided hippocampi were stored in -80 °C for gene expression study, and the left-sided hippocampi were fixed in 10% neutral buffered formalin, processed and embedded in paraffin wax for histological staining and immunohistochemistry.

### Quantitative real-time polymerase chain reaction (qRT-PCR)

Total RNA was extracted using GeneJET RNA Purification Kit (Thermo Scientific, IL, USA) according to the manufacturer's instructions. RNA was then reverse-transcribed into cDNA using Super Script III First Strand cDNA Synthesis Kit (Thermo Scientific, IL, USA). qRT-PCR was performed using SensiFAST™ SYBR® No-ROX Kit (Bioline USA Inc. USA) for the LC3B gene using the primer sequences shown in Table 2, according to the manufacturer's instructions. Beta-actin was used as an endogenous reference control. All samples were quantified using Applied Biosystems™ 7500 Real-Time PCR System. The 2−^ΔΔCt^ method was conducted for analysis of gene expression levels.

**Table 2:** Primers sequences used in qRT-PCR (Lu et al. 2015)

Name	Sequence
LC3B Forward	5′-GGAGATCTCGCAGGCCTAT-3′
LC3B Reverse	5′-GGCCAGATGTTCATCCACTT-3′
Beta-actin Forward	5′-CCACAGCTGAGAGGGAAATC-3′
Beta-actin Reverse	5′-TGCCGATAGTAGTAGCCTGA-3′

### Histopathological examination

#### Hematoxylin & eosin (H&E) stain

5-μm thick horizontal sections of rat hippocampus were cut, mounted on slides and stained by H&E to examine the histological architecture of CA1 neurons;

#### Nissl stain

20-μm thick sections were used to assess effects of treatments on hippocampal neuronal cell survival. The Nissl staining technique was performed as previously described (Kataoka et al. 2013). Briefly, deparaffinized sections were stained with warm 0.1% Cresyl Violet solution for 20 min, rinsed in distilled water and dehydrated with alcohol.

#### Immunohistochemistry

5-μm thick sections were used to assess efficiency of new autophagosome biogenesis, using LC3B antibody (rabbit polyclonal antibodies for LC3B, 1:200) according to the manufacturer's instructions; P62 immunohistochemistry, to detect somatic P62 aggregates, using P62 antibody (rabbit polyclonal P62 antibody, 1:100) according to the manufacturer's instructions. According to the Avidin Biotin Peroxidase technique (Scult et al. 2019), the deparaffinized sections were incubated with the primary antibody for the time recommended by the manufacturer. Unbound primary antibody was washed out, and slides were then incubated with the secondary antibody, counter-stained with hematoxylin and visualized using diaminobenzidine. Negative control sections, done after omitting primary antibodies, showed no specific labeling.

#### Quantitative image analysis

Hippocampal CA1 region was assessed qualitatively using light microscope (Olympus, CX21FS1, Tokyo, Japan). Subsequently, a ToupCam digital camera (SCMOS05000KPB) was used to obtain high-quality digital images for quantitative analysis and data interpretation. Nissl-positive neuronal cell numbers were counted using ImageJ 1.50i software (Schneider et al. 2012). The positive cell count/mm^2^ was averaged from at least twenty sections per group.

For immunostaining, the two regions of interest “stratum pyramidale (SP) and stratum radiatum (SR) of Cornu Ammonis 1 (CA1)” were defined using the rat brain atlas of (Paxinos and Watson 2013). LC3B expression in the stratum radiatum was expressed as the LC3B immunopositive area fraction (%); the ratio of LC3B immunopositive area to the entire area of stratum radiatum (extending 400-450 μm deep to stratum pyramidale) for each IHC image. LC3B expression in the stratum pyramidale was expressed as the diaminobenzidine mean staining intensity normalized to nuclei count (in the representative stratum pyramidale) for each IHC image (Crowe and Yue 2019). Since images were in a RGB format, the ‘Color Deconvolution’ plugin in FIJI/ImageJ was used to deconvolve the color information, followed by conversion to an 8-bit format, to enhance the dynamic range of the signal. At least 20 random microscopic fields/group were analyzed using FIJI/IMAGE J v1.53d software (Media Cybernetics, Inc., Rockville, MD, USA) (Schindelin et al. 2012). P62 expression in the stratum pyramidale was expressed as the percentage (%) of cells detected with P62 immunolabeling using the image analysis software, QuPath (Bankhead et al. 2017).

### Statistical analysis

Statistical analysis was done using SPSS (Statistical Package for Social Science) program for windows v 24. Charts were constructed using Microsoft Excel program and data are presented as mean ± SEM. After *Shapiro–Wilk* normality test, *one-way analysis of variance (ANOVA)* was used to evaluate results of behavioral testing, real-time PCR and quantitative histopathology. Significant effects of different treatments were compared using the *Tukey* post hoc test. *Paired samples t-test* was also used analyze results of the three-chamber social approach and light/dark transition tests. For all comparisons, the statistical significance was set at p < 0.05. The correlations between quantitative LC3B gene/immunohistochemical expression and Nissl-positive cell count/mm^2^ were analyzed using *Pearson's r*. p values < 0.001 were considered statistically significant.

Study sample size was determined by power analysis calculated using the G*Power software (version 3.1.9.7). Based on the variance estimates obtained from our pilot experiments, we hypothesized a considerably large effect size (Cohen’s f = 0.8) when comparing the four study groups as regards sociability indices (time spent sniffing novel rat and social preference index). In a one-way ANOVA study, a total sample size of 24 (6 rats are obtained from each of the 4 groups) achieves 86% power to detect differences among the means.

## Results

### Effect of postnatal metformin/risperidone therapy on ASD-like behaviors of male offspring prenatally-exposed to VPA

Prenatal exposure to VPA is associated with neurodevelopmental deficits, relevant to autism in laboratory animals. In this study, we examined how rats prenatally exposed to VPA might benefit from postnatal treatment with metformin or risperidone. When VPA was injected to pregnant females at E12.5, their adolescent male offspring displayed impaired social behaviors, aberrant grooming activities and elevated anxiety, compared to normal controls. One month treatment (from P21-P51) with either metformin or risperidone significantly improved such deficits, compared to VPA-untreated group.

#### The three chambers social approach test

**Fig. 1:**
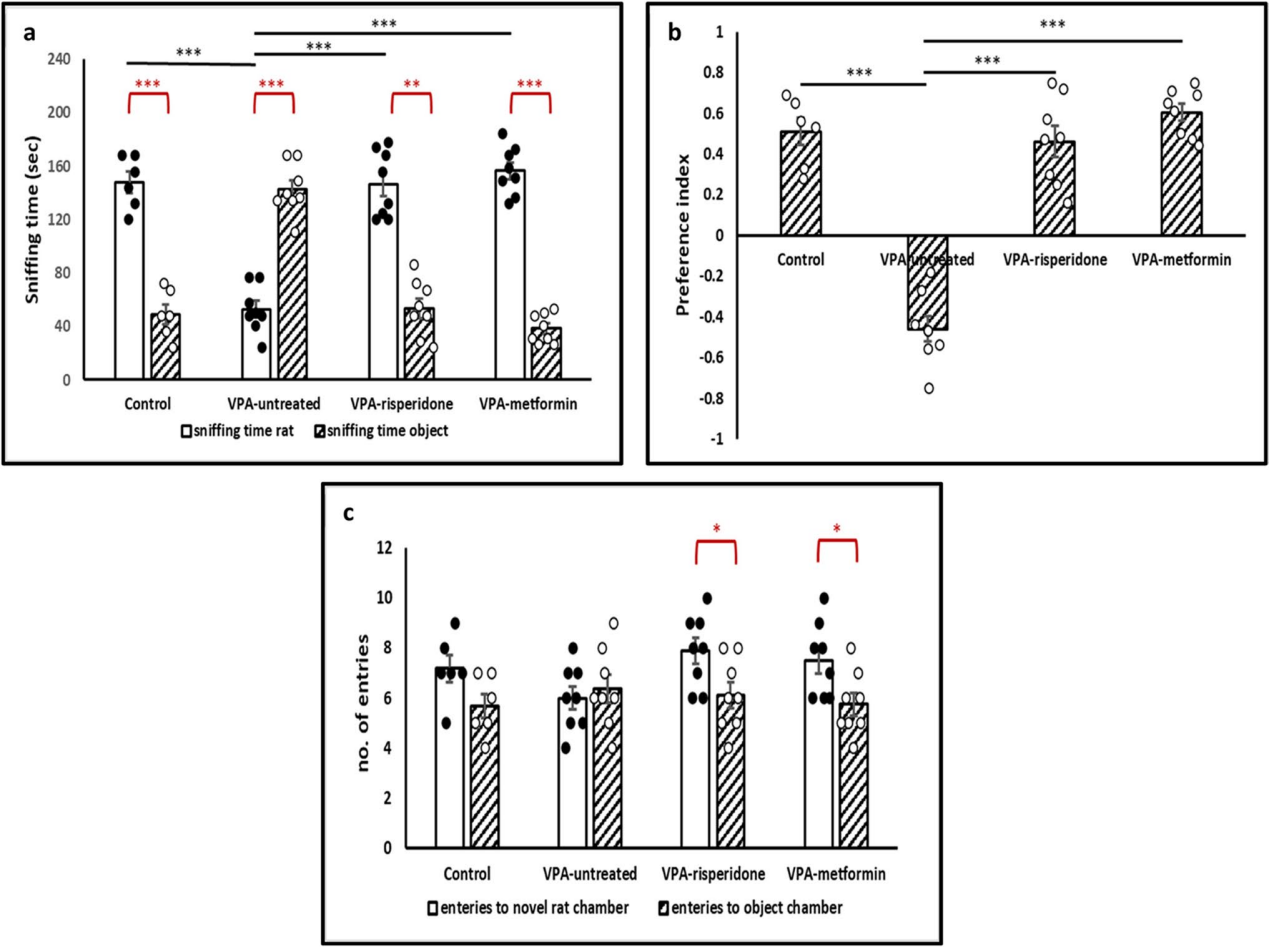
The effect of risperidone/metformin treatment on social deficits in male offspring prenatally exposed to VPA, using the three-chamber social approach test (**a**) Compared to normal control group, male rats of the VPA-untreated group spent significantly less time sniffing a novel social stimulus (rat). risperidone/metformin treatment efficiently rescued social interaction with the novel rat. (**b**) Compared to Normal, VPA-untreated rats displayed markedly reduced preference index that was efficiently improved by risperidone/metformin treatment. (**c**) No significant difference in total entries to either chamber was observed between all groups. Data are presented as the means ± SEM (n = 6-8). Statistical analyses were performed using one-way *ANOVA*, followed by *Tukey’s* multiple comparison test & paired samples *t-test.* ∗p < 0.05, ∗∗p < 0.01, and ∗∗∗p < 0.001

Results of *the paired samples t-test* revealed that control males spent significantly more time face-sniffing the novel rat as compared to the time spent sniffing the nonsocial novel object. E12.5 exposure to VPA significantly reduced preference for the novel rat compared to the novel object (Figure 1a, Table 3). However, both metformin and risperidone did rescue the sociability deficit observed in the prenatally-exposed VPA rats as confirmed by the marked increase in the time spent face-sniffing the novel rat compared to that spent sniffing the object (Figure 1a, Table 3). Both treated groups also displayed entries into the novel rat chamber that were significantly more than those into the object chamber, further validating restoration of the characteristic rodent social behavior **(**Figure 1c, Table 3).

**Table 3:** The effect of risperidone/metformin treatment on social deficits, repetitive behaviors and anxiety in male offspring prenatally exposed to VPA

			Control	VPA-untreated	VPA-risperidone	VPA-metformin	*One-way ANOVA* *F (3,26)*
The 3-chamber social approach	Time sniffing novel rat (sec)		148 ± 8	52.8 ± 6.27^***^	146.55 ± 8.83^$$$^	156.6 ± 6.38^$$$^	*F = 45.57* *P < 0.001*
	Time sniffing novel object (sec)		49.2 ± 7.43	142.5 ± 6.74^***^	53.7 ± 7.51^$$$^	38.4 ± 3.88^$$$^	*F = 57.71* *P < 0.001*
		*Paired samples t-test*	*t = 8.02* *df = 5* *p < 0.001*	*t = -7.46* *df = 7* *p < 0.001*	*t = 5.93* *df = 7* *p = 0.001*	*t = 12.41* *df =7* *p < 0.001*	
	No. of entries into novel rat chamber		7.17 ± 0.54	6 ± 0.49	7.88 ± 0.52	7.5 ± 0.53	*F = 2.64* *P = 0.07*
	No. of entries into novel object chamber		5.67 ± 0.49	6.38 ± 0.56	6.13 ± 0.52	5.75 ± 0.45	*F = 0.41* *P = 0.75*
		*Paired samples t-test*	*t = 1.7* *df = 5* *p = 0.15*	*t = -0.5* *df = 7* *p = 0.63*	*t = 2.82* *df = 7* *p = 0.03*	*t = 3.56* *df = 7* *p = 0.009*	
	Preference index		0.51 ± 0.07	-0.46 ± 0.06^***^	0.46 ± 0.08^$$$^	0.60 ± 0.04^$$$^	*F = 64.85* *P < 0.001*
Spontaneous grooming	Duration of total grooming (sec)		38 ± 4.44	81.63 ± 4.99^***^	41 ± 3.13^$$$^	42.75 ± 4.2^$$$^	*F = 24.21* *P < 0.001*
	No. of complete grooming bouts		2.83 ± 0.6	4.63 ± 1.05	3.75 ± 0.53	3.38 ± 0.78	*F = 0.87* *P = 0.47*
	% of incorrect transitions		56.22 ± 4.56	40.53 ± 2.84^*^	59.63 ± 3.57^$$^	57.79 ± 3.99^$^	*F = 5.94* *P = 0.003*
Light/dark test	Time spent in light compartment		108.904 ± 7.6	31.45 ± 6.47^***^	86.71 ± 10.01^$$$^	131.58 ± 2.5^$$$@@^	*F = 37.48* *P < 0.001*
	Time spent in dark compartment		191.097 ± 7.6	268.55 ± 6.47^***^	213.29 ± 10.01^$$$^	168.42 ± 2.5^$$$@@^	*F = 37. 48* *P < 0.001*
		*Paired samples t-test*	*t = -5.41* *df = 5* *p = 0.003*	*t = -18.32* *df = 7* *p < 0.001*	*t = -6.32* *df = 7* *p < 0.001*	*t = -7.38* *df = 7* *p < 0.001*	
	No. of transitions		3.83 ± 0.48	6.13 ± 0.7	4 ± 0.46	5.5 ± 0.57	*F = 3.81* *P = 0.02*

Data are presented as the means ± SEM (n = 6-8). Statistical analyses were performed using one-way *ANOVA*, followed by *Tukey’s* multiple comparison test or paired samples *t-test* (when appropriate). ^*^p < 0.05, ^**^p < 0.01, and ^***^p < 0.001 *vs* Control; ^$^p < 0.05, ^$$^p < 0.01, and ^$$$^p < 0.001 *vs* VPA-untreated; ^@^p < 0.05, ^@@^p < 0.01, and ^@@@^p < 0.001 *vs* VPA-risperidone

The social preference index was also assessed across the four groups. *One-way ANOVA* indicated significant difference between the preference indices of different groups. Post-hoc testing then revealed a significant lowering in the preference index of the VPA-untreated group compared to control group. Meanwhile, metformin- and risperidone-treated groups displayed markedly higher social preference indices compared with VPA-untreated group **(**Figure 1b, Table 3). Noteworthy, entries into either lateral chamber were not statistically different among normal control, VPA-untreated and treated groups (Figure 1c, Table 3), indicating that impaired sociability, rather than impaired locomotion, underlies the differences observed in the social preference indices across groups.

#### The spontaneous grooming test

**Fig. 2:**
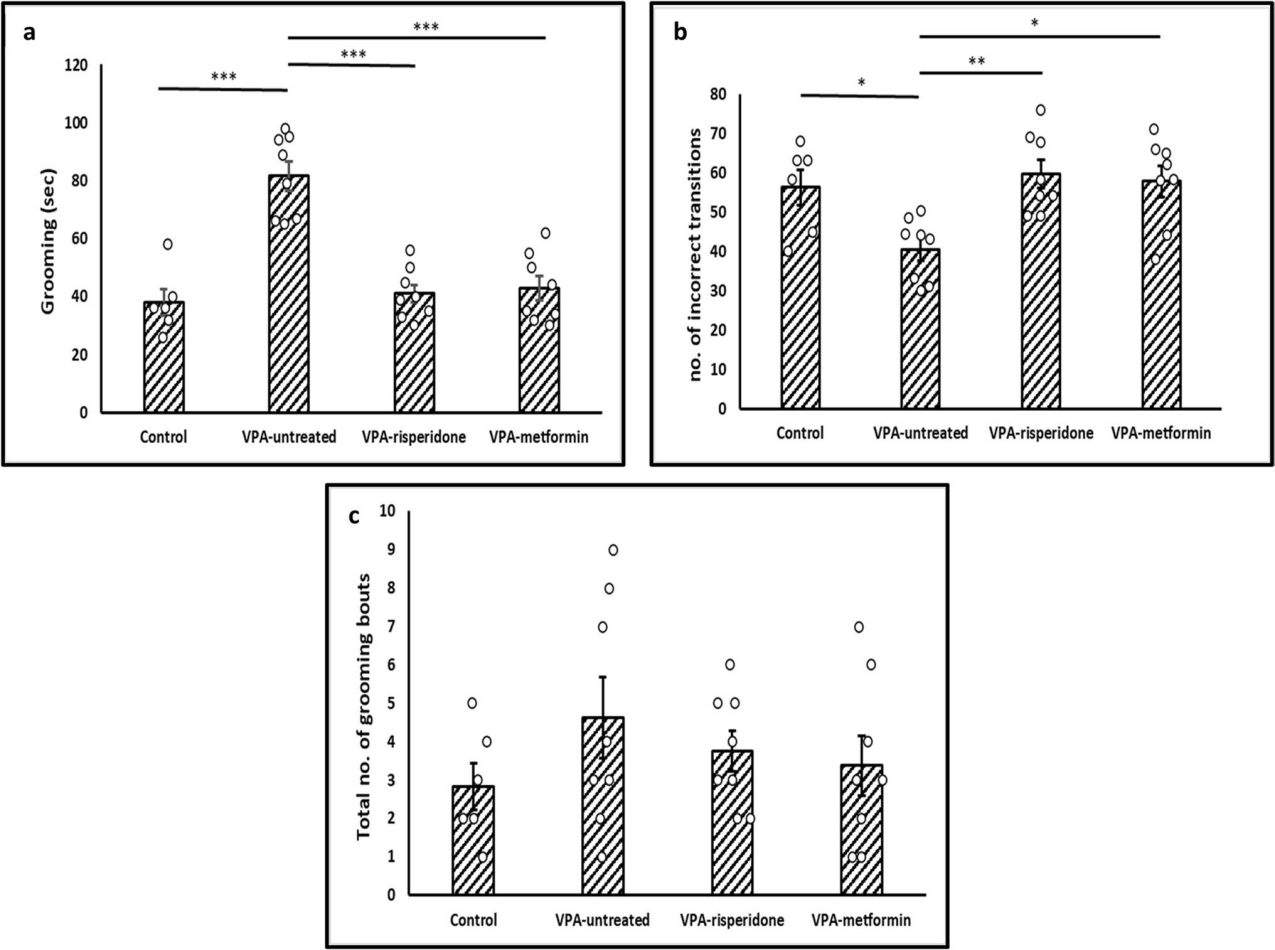
The effect of Risperidone/metformin treatment on repetitive behaviors in male offspring prenatally exposed to VPA, using the spontaneous grooming test (**a**) Male rats of the VPA-untreated group displayed significantly longer durations of self-grooming compared to normal rats. risperidone/metformin treatment efficiently controlled it. (**b**) Compared to Normal, VPA-untreated rats showed markedly decreased proportion of incorrect transitions that was increased by risperidone/metformin treatment. (**c**) No significant difference in the total number of complete grooming bouts was observed between all groups. Data are presented as the means ± SEM (n = 6-8). Statistical analysis was performed using one-way *ANOVA*, followed by *Tukey’s* multiple comparison test. ∗*p* < 0.05, ∗∗*p* < 0.01, and ∗∗∗*p* < 0.001

While the absolute numbers of complete grooming bouts were comparable between normal control and VPA-untreated groups (Figure 2c, Table 3), the total duration of grooming was significantly longer in the VPA-exposed offspring (Figure 2a, Table 3). As expected, postnatal metformin or risperidone treatment efficiently decreased the time spent on self-grooming (Figure 2a, Table 3). One interesting finding is that, although the VPA-untreated group exhibited increased overall grooming activity, the percentage of sequentially-incorrect transitions was significantly lower in this group relative to control (Figure 2b, Table 3). These results suggest that VPA-exposed rats might show a rigorous pattern of repetitive, self-directed behavior, which was markedly improved upon metformin or risperidone treatments (Figure 2b, Table 3).

#### The light/dark test

**Fig. 3:**
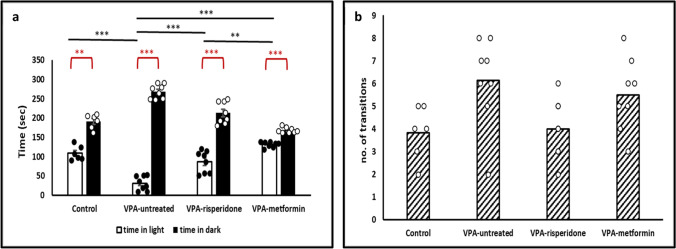
The effect of Risperidone/metformin treatment on anxiety-like behavior observed in the light-dark transitions test (**a**) Compared to normal, prenatal exposure to VPA significantly decreased time spent in the light box, while risperidone/metformin treatment efficiently increased it. (**b**) Number of transitions between the light and dark compartments were not statistically different between all groups. Data are presented as the means ± SEM (n = 6-8). Statistical analyses were performed using one-way *ANOVA*, followed by *Tukey’s* multiple comparison test & paired samples *t-test.* ∗p < 0.05, ∗∗p < 0.01, and ∗∗∗p < 0.001

Consistent with elevated anxiety levels, VPA-untreated offspring displayed marked fear of the brightly-lit arena and spent significantly shorter duration in light compared to normal controls (Figure 3a, Table 3). There is always debate about whether anxiety is best conceptualised as being derived of, or co-morbid to, ASD. In either instance, the high level of anxiety-like behavior observed in our prenatal VPA-exposed rats further confirms the face validity of the adopted model.

Alleviation of anxiety in both metformin- and risperidone-treated groups was confirmed by the statistically significant increase in the time spent in the light compartment (Figure 3a, Table 3). Regarding the number of transitions between light and dark compartments, no statistically significant difference was found among different groups.

### Effect of postnatal metformin/risperidone therapy on the histopathological changes seen in the hippocampi of male offspring prenatally-exposed to VPA

**Fig. 4:**
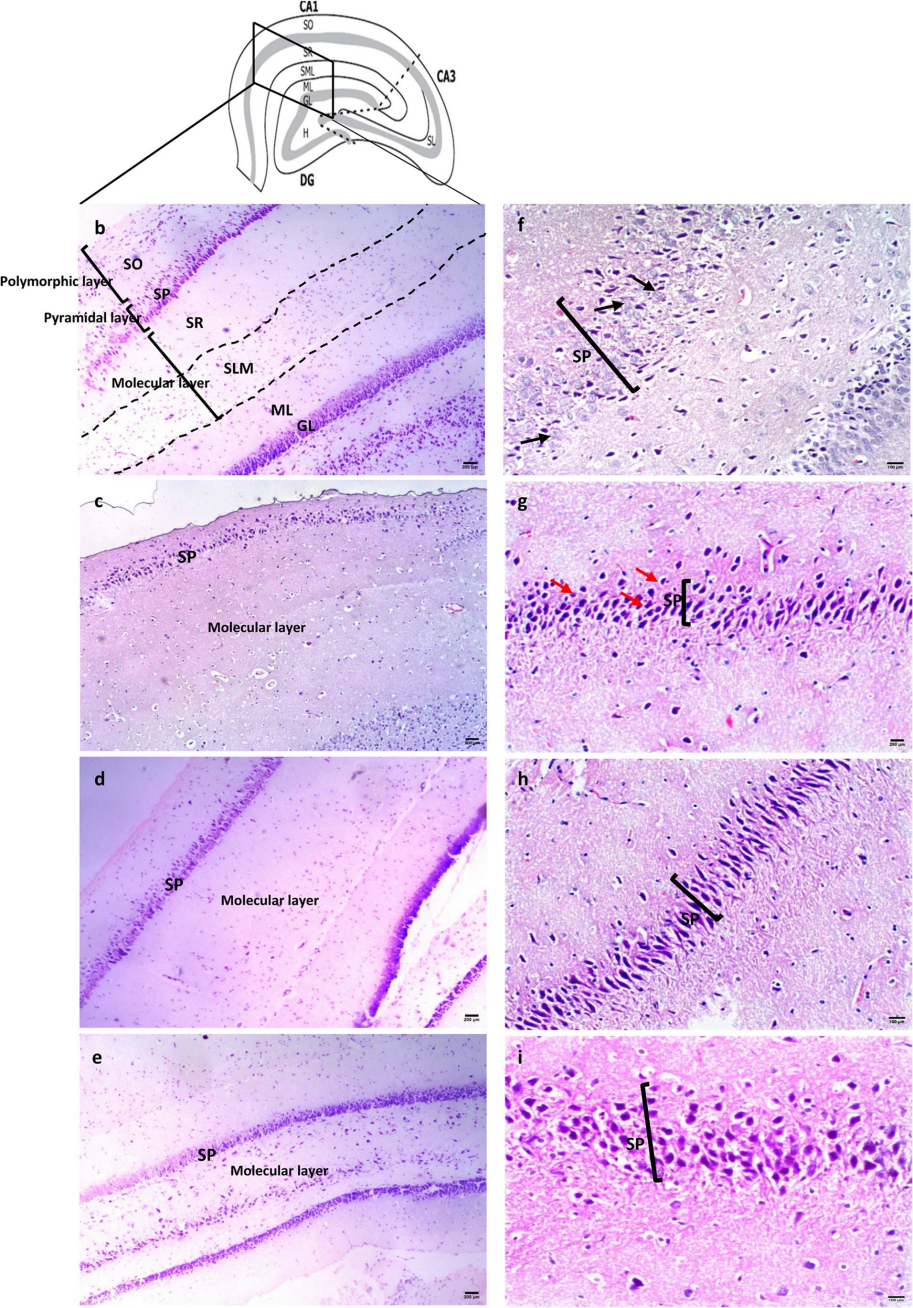
The effect of risperidone/metformin treatment on hippocampal CA1 histopathology (**a**) Diagram of rat hippocampus. Dotted lines separate CA1 from CA3 and CA3 from denture gurus (DG) (Jiang and Swann 1997). SO; stratum oriens, SP; stratum pyramidale, SR; stratum radiatum, SLM; stratum lacunosum moleculare; ML; molecular layer of dentate gurus, GL; granular cell layer, H; hilus of dentate gurus, SL; stratum lucidum. (**b** & **f**) Control group, (**c** & **g**) VPA-untreated group, (**d** & **h**) VPA-risperidone group, (**e** & **i**) VPA-metformin group. H&E staining showed decreased pyramidal neuronal numbers with wide intercellular spaces in the VPA-untreated group. Compared to VPA-untreated and risperidone-treated groups, metformin markedly increased pyramidal cell numbers with increased cytoplasmic processes. Black arrows; normal triangular shaped pyramidal neurons with large vesicular nuclei and prominent cytoplasmic processes, red arrows; shrunken pyramidal neurons with less cytoplasmic processes. Scale bar: 200 μm for (**b**,**c**,**d**,**e**) and 100 μm for (**f**,**g**,**h**,**i**)

Microscopic examination of H&E-stained hippocampus revealed that hippocampus proprius is composed of three regions: Cornu Ammonis 1 – 3 (CA1–CA3) and the dentate gurus (DG). CA1 is roughly divided into three layers: Polymorphic (SO, stratum oriens), pyramidal (SP, stratum pyramidale) and molecular layers (SR, stratum radiatum & SLM; stratum lacunosum moleculare), as shown in Figure 4a and b. Normal CA1 pyramidal neuronal cell bodies are described as large triangular shaped cells with large vesicular nuclei and prominent cytoplasmic processes (Figure 4f).

Microscopic examination of VPA-untreated group revealed sparse arrangement of CA1 pyramidal cells with increased intercellular gaps and reduction in cell numbers (Figure 4c). Pyramidal cells showed shrunken darkly stained nuclei and less cytoplasmic processes (Figure 4g). The glial astrocytic cells within the molecular and polymorphic layers showed marked reduction in number with widened intercellular gaps and pyknotic nuclei. Microscopic examination of hippocampal sections from risperidone-treated group revealed mild focal increase in pyramidal neuron numbers in the CA1 region (Figure 4d), with evidence of neuronal morphological improvement as regard nuclear and cytologic features (Figure 4h). On the other hand, microscopic examination of the CA1 regions from metformin-treated group revealed marked increase in pyramidal cell numbers, along-with restoration of normal neuron morphology (Figure 4e and i).

**Fig. 5:**
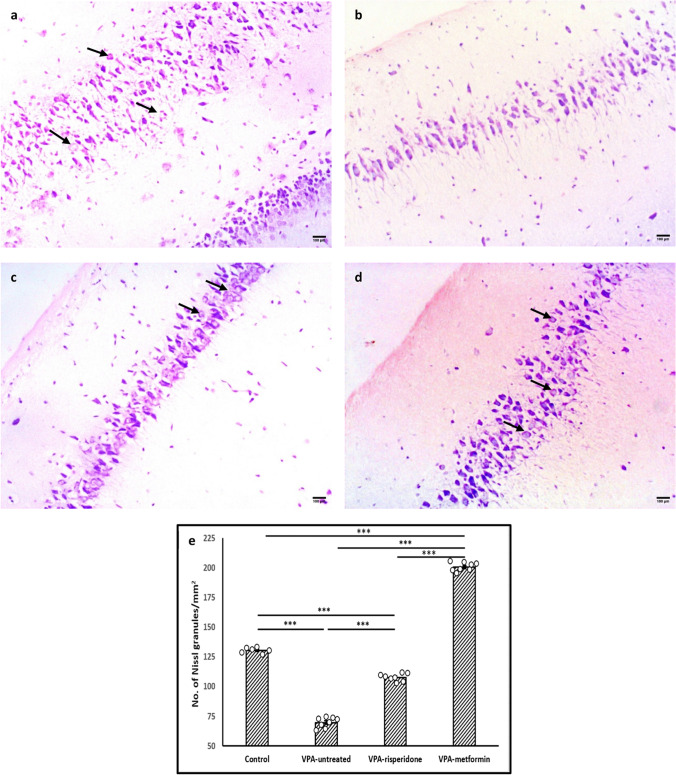
The effect of risperidone/metformin treatment on Nissl-positive neuronal cell numbers Metformin treatment efficiently supported neuronal survival in the hippocampi of male offspring prenatally exposed to VPA. Nissl staining revealed typical pyramidal neuronal loss and damage in the hippocampal CA1 region of VPA-untreated rats. Compared to VPA-untreated and risperidone-treated groups, metformin efficiently suppressed neuronal loss. Black arrows; surviving neurons with rounded pale-stained nuclei. (**a**) Control group, (**b**) VPA-untreated group, (**c**) VPA-risperidone group, (**d**) VPA-metformin group, (**e**) surviving cell densities were represented on a graph by counting Nissl-positive cells per mm^2^. Scale bar: 100 μm. Bars represent mean values ± SEM (n = 6-8). Statistical analysis was performed using one-way *ANOVA*, followed by *Tukey’s* multiple comparison test. ∗p < 0.05, ∗∗p < 0.01, and ∗∗∗p < 0.001

Microscopic assessment of neuronal cell numbers using Nissl-staining method showed that prenatal VPA exposure at E12.5 resulted in neuronal cell loss in the pyramidal layer of rat hippocampi (Figure 5a and b). Significant decrease in the density of Nissl-positive neurons was evident in the CA1 region of VPA-untreated group when compared to normal one (*F (3,26)= 1969.8, p<0.001*, Figure 5e). Meanwhile, tissue sections from both metformin- and risperidone-treated groups displayed significant increase in Nissl-positive neurons density, compared to VPA-untreated group (Figure 5c, d and e). Increments in the metformin-treated group were statistically significant compared to those observed in the risperidone-treated group (Figure 5e).

### Effect of postnatal metformin/risperidone therapy on Autophagy markers in the hippocampi of male offspring prenatally-exposed to VPA

**Fig. 6:**
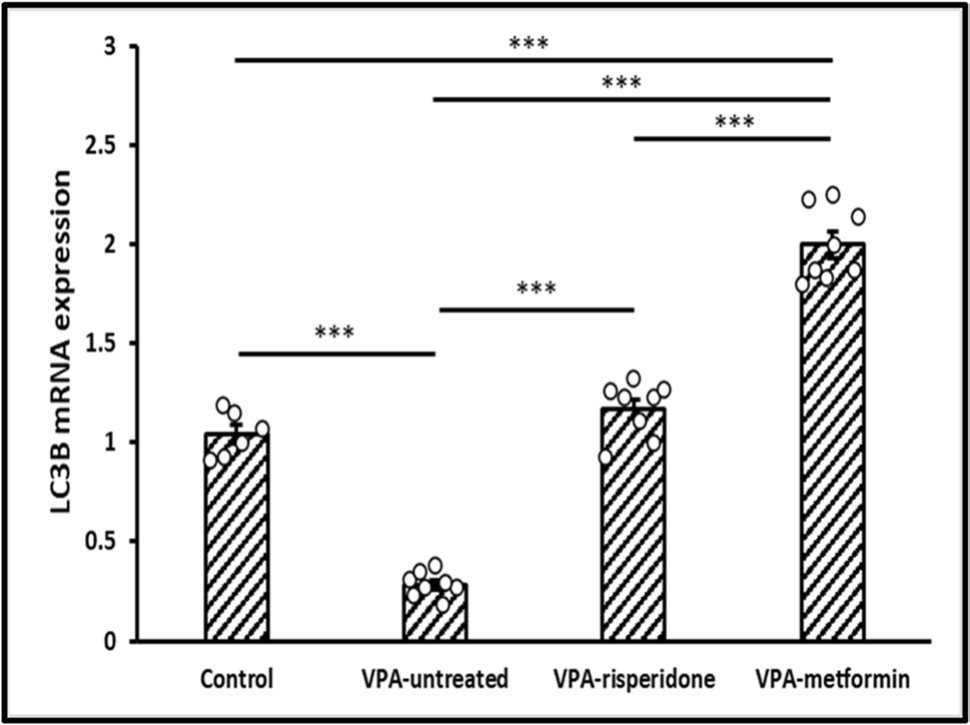
The effect of risperidone/metformin treatment on hippocampal LC3B mRNA expression in male offspring prenatally exposed to VPA Metformin treatment markedly unregulated LC3B mRNA expression in the hippocampi of male offspring prenatally exposed to VPA, compared to VPA-untreated and risperidone-treated groups. Data are presented as the means ± SEM. n = 6-8. Statistical analysis was performed using one-way *ANOVA*, followed by *Tukey’s* multiple comparison test. ∗*p* < 0.05, ∗∗*p* < 0.01, and ∗∗∗*p* < 0.001

**Fig. 7:**
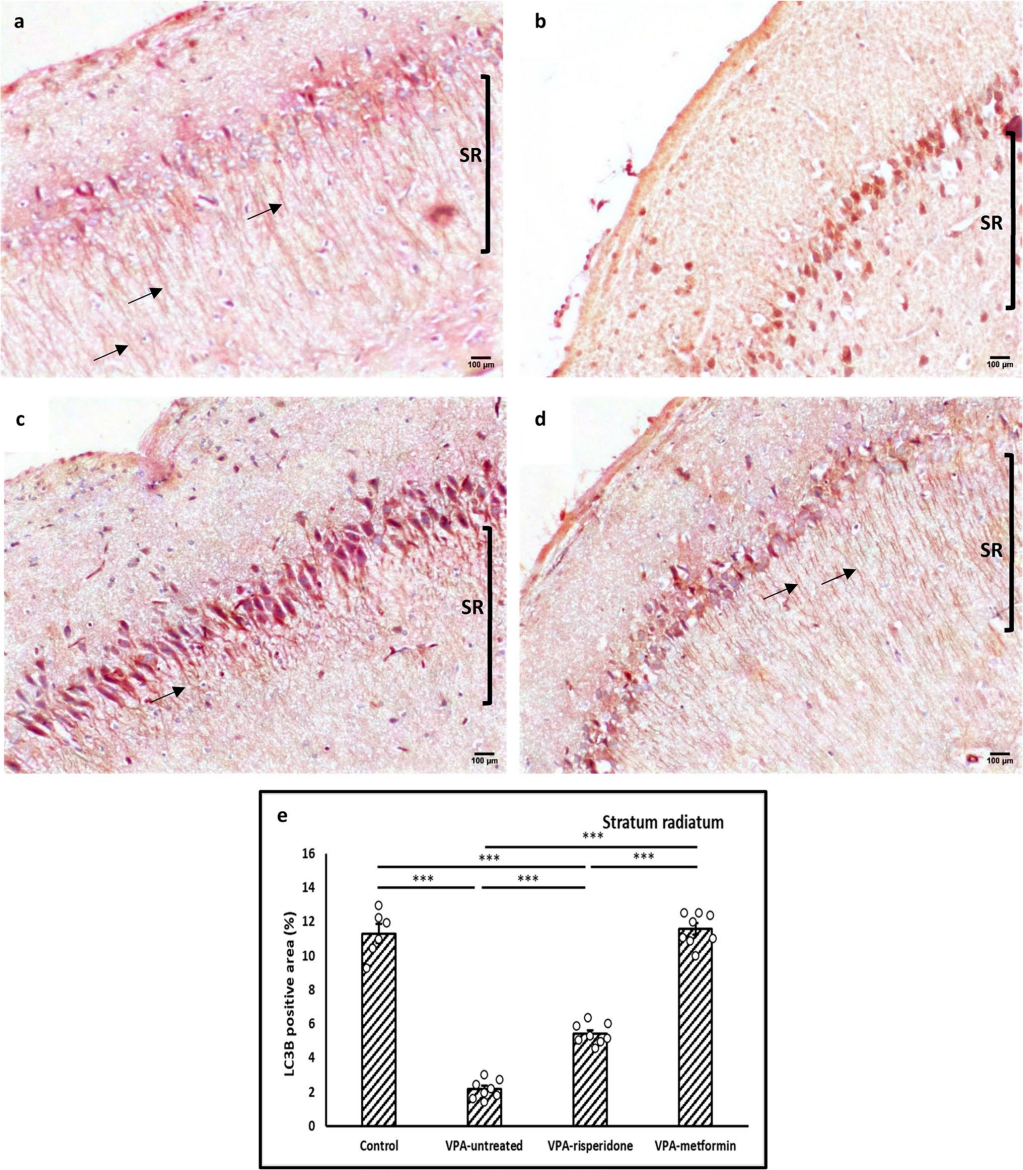
Representative immunohistochemical images of the hippocampal CA1 region immunolabeled with LC3B antibody Metformin treatment efficiently unregulated LC3B expression in the stratum radiatum of male offspring prenatally exposed to VPA. Black arrows; LC3B punctuate immunostaining localized to pyramidale neuronal dendritis. (**a**) Control group, (**b**) VPA-untreated group, (**c**) VPA-risperidone group, (**d**) VPA-metformin group, (**e**) a graph showing LC3B immunopositive area fraction (%) calculated as the ratio of LC3B immunopositive area to the entire area of stratum radiatum (extending 400-450 μm deep to stratum pyramidale) for each IHC image. Scale bar: 100 μm. Bars represent mean values ± SEM (n = 6-8). Statistical analysis was performed using one-way *ANOVA*, followed by *Tukey’s* multiple comparison test. ∗*p* < 0.05, ∗∗*p* < 0.01, and ∗∗∗*p* < 0.001

We assessed autophagosome formation indirectly by assessing gene and protein expression of LC3B. First, LC3B mRNA expression was markedly diminished in the hippocampi of VPA-untreated group relative to the control group (*F (3,26)= 215.39, p<0.001,* Figure 6). Second, immunohistochemical examination of LC3B punctuate expression and distribution in CA1 pyramidal neurons revealed poor delineation of neuronal dendrites in the VPA-untreated group, compared to the well-defined immunostained punctuated dendrites of the normal control group (*F (3,26)= 211.004, p<0.001,* Figure 7a, b and e), indicating reduced autophagosome biosynthesis in the VPA-untreated offspring. In the hippocampal pyramidal neurons, autophagosomes form mainly in dendrites and are then transported to the soma for degradation. LC3B punctate immunostaining in dendrites represents a clue for the newly formed LC3B-labeled autophagosomes.

We next assessed the autophagic flux by measuring abundance of P62 (an adapter protein whose levels are indicators of its lysosomal degradation) in CA1 neurons. P62 was markedly increased in hippocampal sections from VPA-untreated group vs. normal control group (*F (3,26)= 29.04, p<0.001,* Figure 9a, b and e), primarily in the somata, indicating that autophagic flux was down-regulated in the hippocampi of rats prenatally exposed to VPA.

Consistent with enhanced autophagic activity, significant increase in the dendritic LC3B expression (Figure 7c, d and e), along with decreased that of somatic P62 (Figure 9c, d and e), were observed in the CA1 neurons of rats treated with metformin or risperidone, compared to VPA-untreated group. Interestingly, increments in dendritic LC3B and decrements in P62 somatic aggregates were statistically significant in the metformin-treated offspring compared to those observed in the risperidone-treated ones (Figure 7e and 9e). Correlation studies have confirmed the strong positive associations between Nissl-positive neuronal density and both LC3B mRNA/dendritic protein expression (*r= 0.942, p<0.001; r= 0.866, p<0.001, respectively*, Figure 10a and b, Table 4), among all tested animals.

**Table 4.** Pearson’s correlation between quantitative histopathological parameters and autophagy markers

		No. of Nissl granules/mm^2^	LC3B mRNA expression	Dendritic LC3B expression	P62 % positive cells
No. of Nissl granules/mm^2^	Pearson Correlation Sig. (2-tailed)		0.942 P < 0.001	0.866 P < 0.001	-0.567 P = 0.001
LC3B mRNA expression	Pearson Correlation Sig. (2-tailed)			0.787 P < 0.001	-0.5 P = 0.005
Dendritic LC3B expression	Pearson Correlation Sig. (2-tailed)				-0.813 P < 0.001

Strength of correlation is expressed as *Pearson’s r,* with p < 0.001 were considered statistically significant

**Fig. 8:**
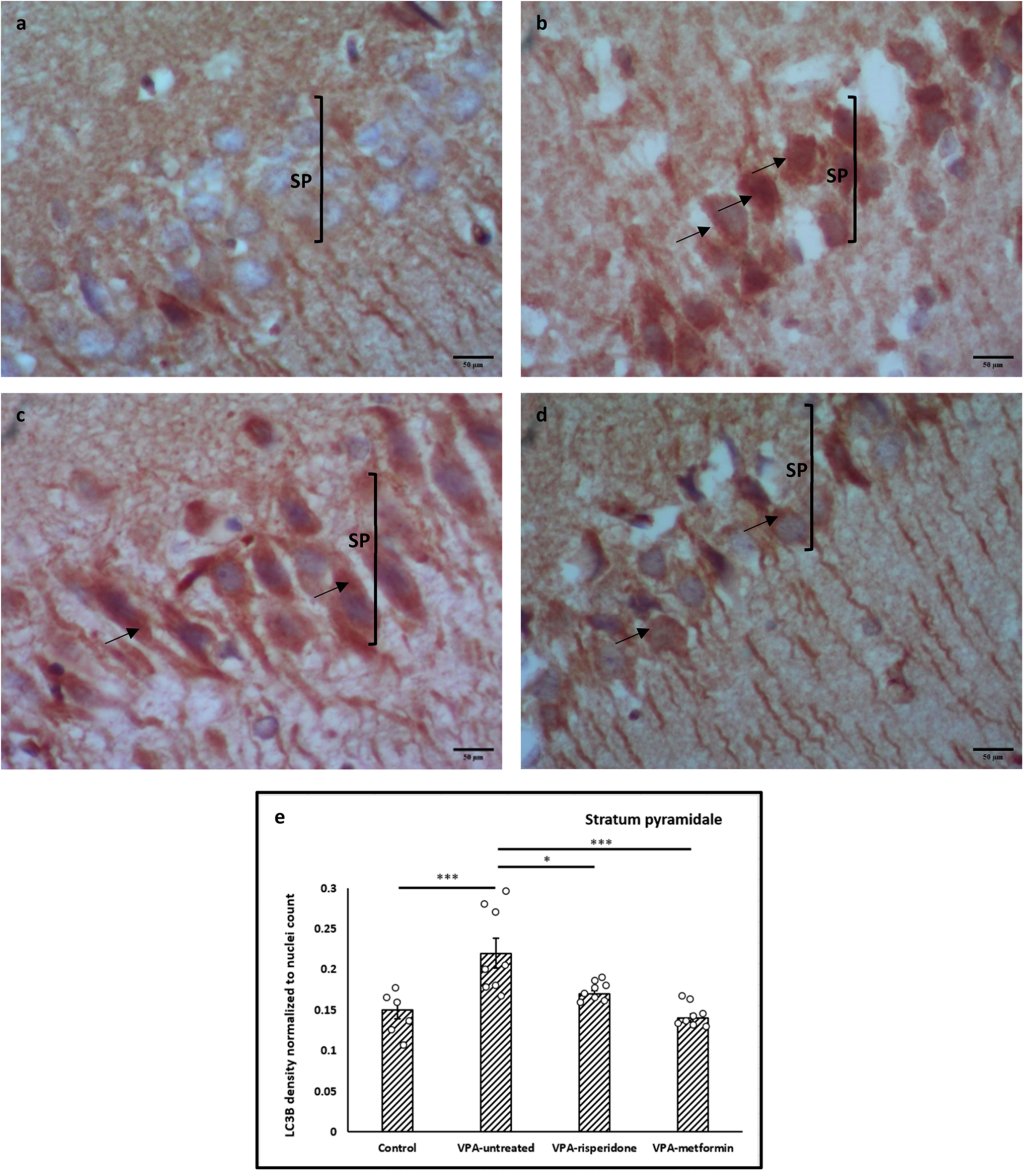
Representative immunohistochemical images of the CA1 stratum pyramidale immunolabeled with LC3B antibody Black arrows; cytoplasmic and nuclear LC3B immunostaining. (**a**) Control group, (**b**) VPA-untreated group, (**c**) VPA-risperidone group, (**d**) VPA-metformin group, (**e**) a graph showing LC3B expression in stratum pyramidale expressed as mean staining intensity normalized to nuclei count. Scale bar: 50 μm. Bars represent mean values ± SEM (n = 6-8). Statistical analysis was performed using one-way *ANOVA*, followed by *Tukey’s* multiple comparison test. ∗p < 0.05, ∗∗p < 0.01, and ∗∗∗p < 0.001

Noteworthy, on analyzing LC3B optical density in the CA1 pyramidal neuronal somata, a significant increase was observed in the cell bodies of VPA-untreated group, compared to control (*F(3,26)= 10.88, p<0.001,* Figure 8a, b and e). Otherwise, a significant decrease in LC3B expression was noted in the soma of neurons belonging to both treated groups, compared to VPA-untreated one (Figure 8c, d and e).

**Fig. 9:**
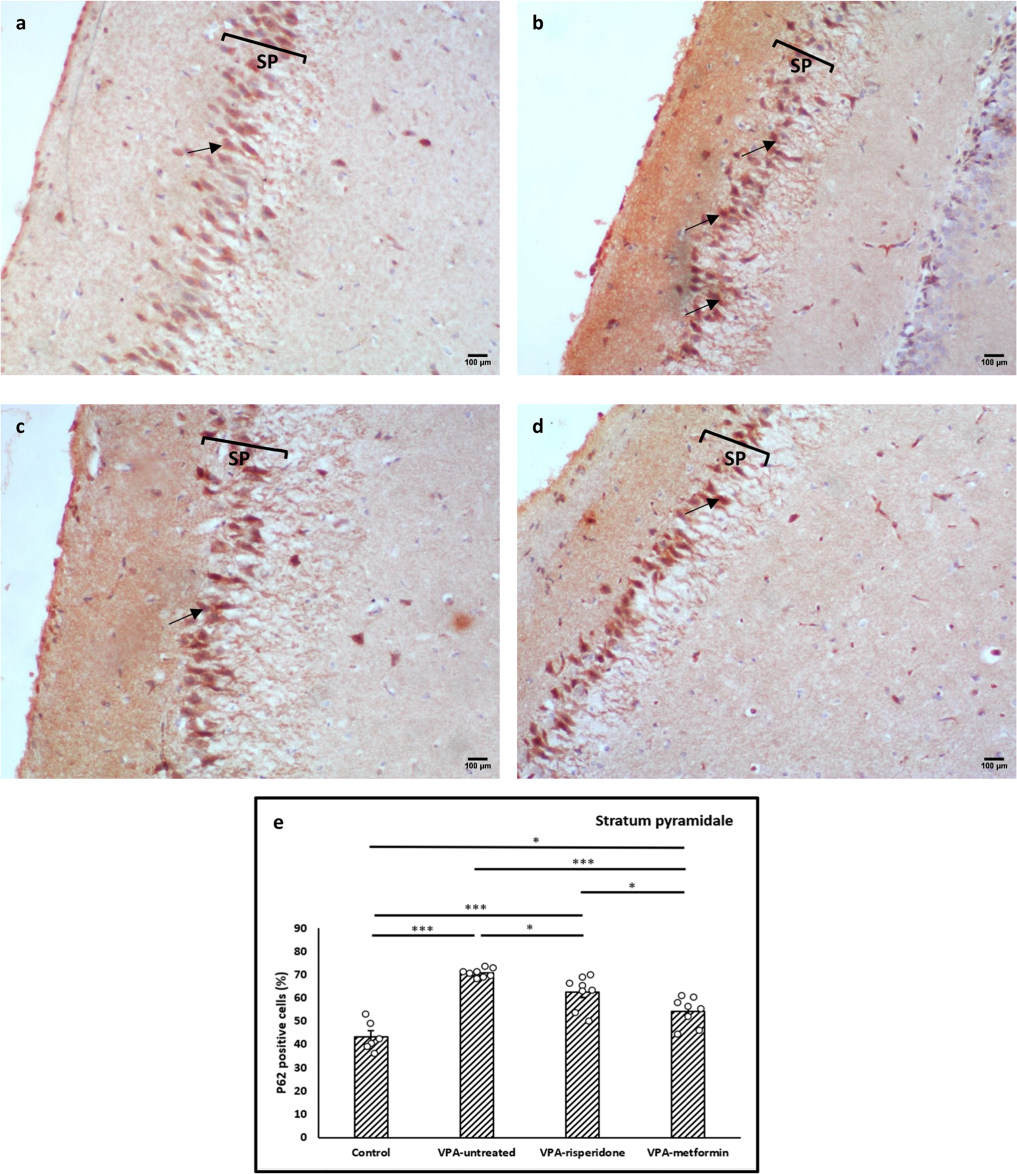
Representative immunohistochemical images of the hippocampal CA1 region immunolabeled with P62 antibody Metformin treatment efficiently reduced P62 protein aggregates in the CA1 pyramidal cell bodies of male offspring prenatally exposed to VPA. (**a**) Control group, (**b**) VPA-untreated group, (**c**) VPA-risperidone group, (**d**) VPA-metformin group, (**e**) a graph showing percent of P62 immunopositive cells (%). Scale bar: 100 μm. Bars represent mean values ± SEM (n = 6-8). Statistical analysis was performed using one-way *ANOVA*, followed by *Tukey’s* multiple comparison test. ∗*p* < 0.05, ∗∗*p* < 0.01, and ∗∗∗*p* < 0.001

**Fig. 10:**
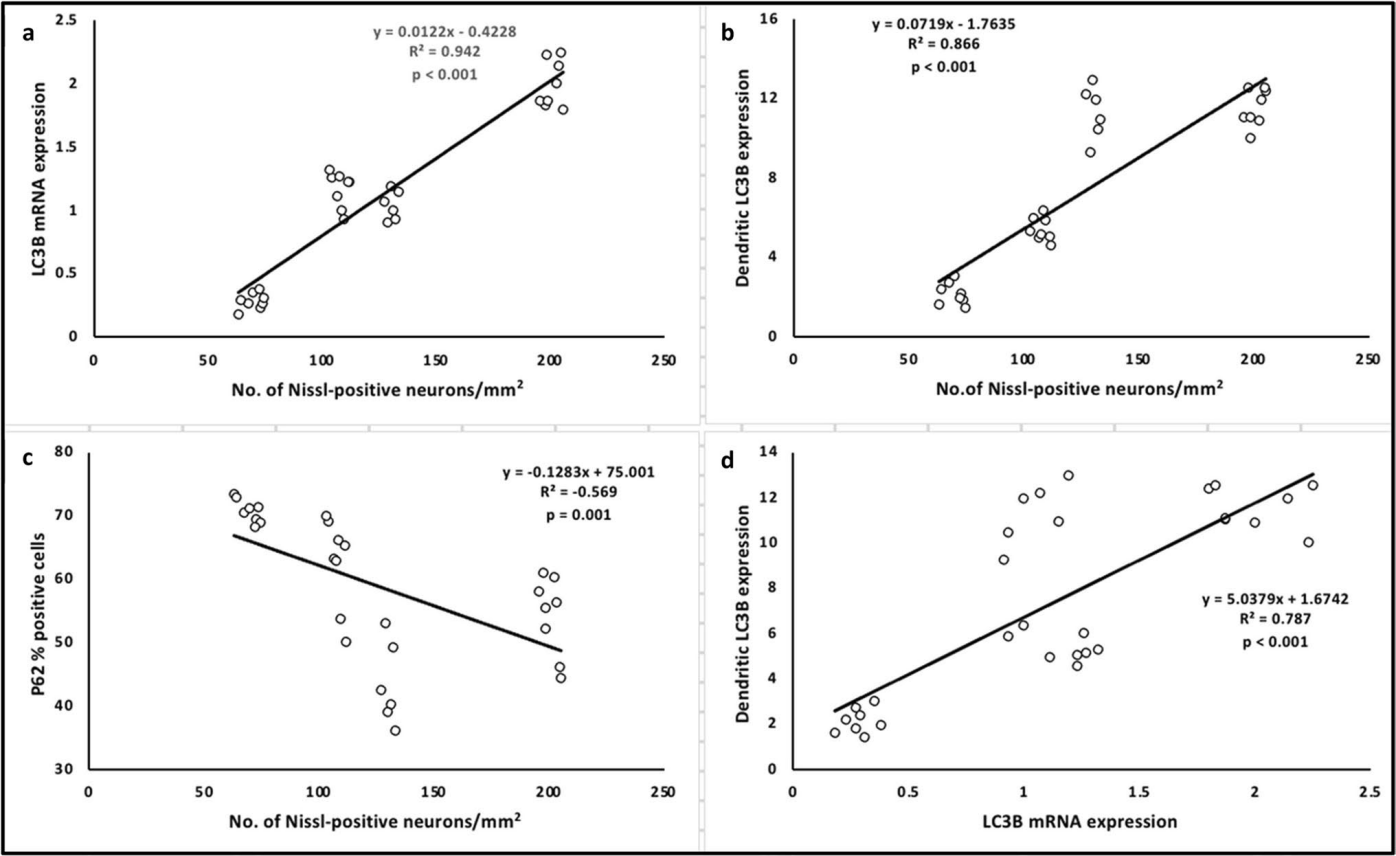
Pearson’s correlation between quantitative histopathological parameters and autophagy markers Correlation between Nissl-positive pyramidal neuron density in the CA1 and hippocampal LC3B mRNA expression (**a**), dendritic LC3B protein expression (**b**) and P62 % immunopositive cells (**c**). Correlation between hippocampal LC3B mRNA expression and dendritic LC3B expression (**d**). Strength of correlation is expressed as *Pearson’s r,* with *p* < 0.001 were considered statistically significant

## Discussion

The main results of the current study reveal that postnatal metformin and risperidone therapy (from P21 to P51) were equally effective in improving ASD-like social deficits, repetitive behaviors and anxiety in adolescent rats prenatally exposed to VPA. Similar to metformin, the efficacy of risperidone was linked to marked enhancement of hippocampus autophagic activity. Our study gives clue, for the first time, that positive modulation of autophagy might underlie the clinical usefulness of risperidone and metformin in ASD.

Over the past two decades, the rodent model of prenatal exposure to VPA has shown to largely recapitulate human ASD behavioral phenotype (Schneider and Przewłocki 2005; Wagner et al. 2006; Nicolini and Fahnestock 2018; Chaliha et al. 2020; Elnahas et al. 2022). In accordance, exposure to neurotoxic dose of VPA (500 mg/kg) during the critical period of embryogenesis (E12.5) elicited ASD-like behavioral abnormalities in the male offspring included in our study. Compared to normal control group, our VPA-untreated rats exhibited impaired sociability (evidenced by lower preference for novel rat); increased grooming activity; and elevated anxiety levels (manifested as increased avoidance of brightly-lit environment).

In the three-chamber social approach test, prenatal VPA exposure resulted in markedly less interaction time with the novel rat (social stimulus). Such social dysfunction is likely caused by, defects in the cognitive functions needed (Schiavi et al. 2019) for proper perception and understanding of socially relevant cues; failure to respond to the changing social circumstances; or may be due to dysfunction of the dopaminergic mesocorticolimbic reward processing a positive impact of the social experience (Pavăl et al. 2017). The hippocampus, in particular, has been recently reframed as an organizer of social information. Impairment in the complex, hippocampal-driven cognitive mapping in ASD would impair one’s understanding of the relationships with external stimuli, leading to difficulties in predicting the actions of social others as well as in assessing the ways one can keep track within a given variable and dynamically changing social environment (Banker et al. 2021).

Improved sociability of our adolescent rats treated with either risperidone or metformin was confirmed by the obviously higher preference to spend time with the novel rat rather than with the object (non-social stimulus). Our results correlate with previous studies (Hara et al. 2017; Wang et al. 2018; Ishola et al. 2020; Román et al. 2021; Deng et al. 2022b; Elnahas et al. 2022)

Object/stranger chamber entries were not statistically different across our VPA-treated and -untreated groups. Similar observation was previously reported with metformin therapy in BTBR mouse model of autism (Wang et al. 2018). According to Wang et al., (2018), non-significant chamber entries highlight improved sociability, rather than locomotion, as a mechanism for the strong preference of the stranger rat seen in metformin/risperidone-treated groups.

For assessment of compulsive-like behaviors, we analyzed rodents’ self-grooming activity as a typical example, when elevated, of a highly stereotyped pattern of movements observed in ASD rodents (Silverman et al. 2010). As expected from similar studies (Hirsch et al. 2018; Ryu et al. 2021), our rats prenatally exposed to VPA spent significantly more time grooming themselves, and this time was effectively reduced by metformin or risperidone therapy.

Anxiety is one important comorbidity in ASD children that might provoke restrictive behaviors and predict a poorer quality of life (Kimura et al. 2020). In line with our results, other studies have also indicated the anxiogenic effects of prenatal VPA treatment (Servadio et al. 2016; Tartaglione et al. 2019; László et al. 2022)

Our findings indicate that both metformin and risperidone therapy had anxiolytic effects when given to postnatally to VPA-exposed male rats. Namely, our results showed that the time spent by the VPA-exposed rats in the light compartment of the light/dark box increased following metformin/risperidone treatment and even reached the normal levels with metformin. Mechanisms by which metformin ameliorates the characteristic ASD behavioral phenotypes remains an interesting question. Down rearrangement of mTOR/mTORC1 signaling in the cerebral cortex (Wang et al. 2018) or cerebellum (Liu et al. 2022), down-regulation of the pro-inflammatory cytokines (NF-κB, IL-17A, and IL-6) in the hippocampus (Deng et al. 2022b), reestablishment of oxide-antioxidant balance and enhancement of cortical and hippocampal cholinergic activity (Ishola et al. 2020), selective abundance of some gut microbiota and/or enhanced serotonin production in the colon (Deng et al. 2022a); were all suggested in preclinical models treated with metformin. In similar context, the favorable behavioral effects of risperidone in ASD were explained by its ability to suppress neuroinflammation (Elnahas et al. 2022), restore redox balance and regulate serotonergic and dopaminergic transmission in the prefrontal cortex and hippocampus (Hara et al. 2017).

Autophagy is a highly conserved cellular degradation pathway that is responsible for recycling long-lived proteins and damaged organelles (Ohsumi, 2014). As a pathologic mechanism, autophagic over-suppression has been reported in both genetic and environmental rodent models of ASD (Lieberman et al., 2020; Qin et al., 2016; Tang et al., 2014; Zhang et al., 2016). Evidence that prenatal exposure to VPA is associated with below normal hippocampus autophagy firstly came from the work of J. Zhang et al. (2016). Others have highlighted the potential of improving ASD behavioral abnormalities upon autophagy induction using inhibitors of mTOR (Qin et al. 2016),

Via disinhibition of unc-51 like autophagy activating kinase 1 (ULK1), mTOR inhibitors can initiate the formation of the U-shaped isolation membranes (phagophores) which are then expanded and sealed forming autophagosomes; a process that involves processing of LC3B. The closed mature autophagosomes then traffic to lysosomes where the autophagic cargo and cargo adaptor, P62, are degraded (Lieberman et al. 2020). Autophagy is a dynamic multi-step process, thus we used LC3B as a biomarker for new autophagosome formation and P62 as an autophagic substrate to verify the lysosome degradation (Klionsky et al. 2012). Gene and immunohistochemical analysis of the two major proteins confirmed suppression of autophagic activity in the hippocampi of VPA-untreated group, while uncovered enhancement of autophagy in the metformin- and risperidone-treated groups. Both treatments markedly upregulated LC3B gene expression and protein abundance in the dendritic branches of CA1 pyramidal neurons, while considerably reduced P62 aggregates in their cell bodies.

Despite not previously described in models of ASD, evidence strongly supports metformin’s autophagy inducing properties, such that numerous AMPK-dependent signaling pathways were implicated including downstream inhibition of mTOR and/or activation of ULK1 (Lu et al. 2021). Evidence for autophagic modulation by risperidone (atypical antipsychotic) is much less clear. While it was shown to induce autophagy in SH-SY5Y neuronal cells (Vucicevic et al. 2014), it exhibited suppressive potential in MC3T3-E1 osteoblast cell line (Zhang et al. 2022). Similar conflicting data were reported for other antipsychotics (Vucicevic et al. 2018; Liu et al. 2019) used in different experimental situations. One explanation was provided by Ibarra-Lecue et al. (2020). They proposed that depending on duration of therapy (acute vs. chronic), experimental setting (in vitro vs. in vivo) and differences in the affinity for Dopamine (D2) receptors, antipsychotics might differentially regulate neuronal Akt/mTOR pathway, and possibly autophagy though this needs further research.

Postmortem histological examinations have confirmed the presence of increased dendritic spine densities in the brains of ASD patients (Tang et al. 2014; Weir et al. 2018). Tang et al., (2014) were the first to describe a mechanistic link between mTOR-driven impaired autophagy and deficient synaptic pruning/elimination in the temporal cortex of ASD patients at different ages. Similar to cortical neurons, biogenesis of autophagosomes in hippocampus pyramidal neurons is spatially initiated in the axon tip and phagophores mature to complete vacuoles as they are retrogradely transported from the axon tip towards the soma. Recently, dendritic autophagy in the hippocampus CA1 pyramidal neurons was deemed indispensable for long-term depression of synaptic strength (Kallergi et al. 2022), a process suggested to underlie developmental excitatory synaptic pruning and cognitive flexibility.

Using an antibody against LC3B, a protein that appears punctate when associated with autophagic membranes, the signal of LC3B puncta in the pyramidal neuronal dendrites of VPA-untreated group disappeared shortly away from the soma, indicating that autophagosomes are scarce in the hippocampal dendrites. Although both metformin and risperidone triggered statistically significant increase in LC3B immuno-positivity, this increase was more prominent and spatially localized to dendrites in the metformin-treated group compared to risperidone-treated one, which might imply importance of autophagic enhancement as a mechanism of metformin’s action in ASD.

Unlike the overall similar behavioral efficacy, histopathological improvements, as well as, neuronal survival were obviously better due to metformin therapy compared to risperidone. Correlation workup has confirmed statistically significant strong positive association between LC3B gene/dendritic protein expression and neuronal survival (density of CA1 Nissl positive neurons), suggesting autophagy enhancement as primarily implicated in metformin’s ability to combat ASD-related hippocampus structural changes.

Supporting findings were drawn from P62 immunohistochemical analysis. Hippocampal P62 aggregates noted in the pyramidal cell bodies of our VPA-untreated group were efficiently cleared by metformin, compared to risperidone-treated group. Similarly, we report an increase in LC3B expression in the pyramidal neuronal somata of VPA-untreated group when compared to the control group. Despite seeming contradictory, such somatic LC3B increments were previously reported by Runwal et al. (2019) when hippocampal autophagosome biogenesis and/or degradation was abrogated, and were attributed to LC3B sequestration to P62 aggregates. Lowered somatic LC3B expression caused by metformin/risperidone therapy is thus readily explained by enhanced autophagic clearance of LC3B sequestrations along with clearance of P62 aggregates. Similar LC3B expression findings were recently reported by Kallergi et al., (2022) upon autophagy induction in the hippocampal pyramidal neurons.

## Conclusion

In summary, the present study shows that male rats prenatally exposed to VPA exhibited multiple ASD-like behavioral abnormalities, along with histopathological changes in their hippocampi that were highly correlated to suppressed autophagic activity. These histopathological aberrations were significantly improved by enhancing hippocampal autophagy with metformin (compared to risperidone). Both drugs comparably alleviated ASD-like behavioral deficits, thus, providing evidence that autophagy enhancement in the hippocampus is a potential therapeutic strategy to improve ASD core symptoms.

## Data Availability

The data that support the findings of this study are available from the corresponding author, [Ramadan NM], upon reasonable request.
